# Identifying potential drug-target interactions based on ensemble deep learning

**DOI:** 10.3389/fnagi.2023.1176400

**Published:** 2023-06-15

**Authors:** Liqian Zhou, Yuzhuang Wang, Lihong Peng, Zejun Li, Xueming Luo

**Affiliations:** ^1^School of Computer Science, Hunan University of Technology, Zhuzhou, China; ^2^School of Computer Science, Hunan Institute of Technology, Hengyang, China

**Keywords:** drug-target interaction, gradient boosting neural network, deep neural network, deep forest, Parkinson's disease, Alzheimer's disease

## Abstract

**Introduction:**

Drug-target interaction prediction is one important step in drug research and development. Experimental methods are time consuming and laborious.

**Methods:**

In this study, we developed a novel DTI prediction method called EnGDD by combining initial feature acquisition, dimensional reduction, and DTI classification based on Gradient boosting neural network, Deep neural network, and Deep Forest.

**Results:**

EnGDD was compared with seven stat-of-the-art DTI prediction methods (BLM-NII, NRLMF, WNNGIP, NEDTP, DTi2Vec, RoFDT, and MolTrans) on the nuclear receptor, GPCR, ion channel, and enzyme datasets under cross validations on drugs, targets, and drug-target pairs, respectively. EnGDD computed the best recall, accuracy, F1-score, AUC, and AUPR under the majority of conditions, demonstrating its powerful DTI identification performance. EnGDD predicted that D00182 and hsa2099, D07871 and hsa1813, DB00599 and hsa2562, D00002 and hsa10935 have a higher interaction probabilities among unknown drug-target pairs and may be potential DTIs on the four datasets, respectively. In particular, D00002 (Nadide) was identified to interact with hsa10935 (Mitochondrial peroxiredoxin3) whose up-regulation might be used to treat neurodegenerative diseases. Finally, EnGDD was used to find possible drug targets for Parkinson's disease and Alzheimer's disease after confirming its DTI identification performance. The results show that D01277, D04641, and D08969 may be applied to the treatment of Parkinson's disease through targeting hsa1813 (dopamine receptor D2) and D02173, D02558, and D03822 may be the clues of treatment for patients with Alzheimer's disease through targeting hsa5743 (prostaglandinendoperoxide synthase 2). The above prediction results need further biomedical validation.

**Discussion:**

We anticipate that our proposed EnGDD model can help discover potential therapeutic clues for various diseases including neurodegenerative diseases.

## 1. Introduction

Identification of Drug-Target Interactions (DTIs) for various diseases is one key step in drug research and development (Peng et al., 2015; Zhang et al., 2019; Chu et al., 2022; Zhou et al., 2022; Liu et al., 2023), however, it is time-consuming, costly, and low-success rate (Dickson and Gagnon, 2004; Kola and Landis, 2017; Peng et al., 2022b; Shen et al., 2022). Drug repositioning (Wang and Zeng, 2013; Liang et al., 2022a; Tian et al., 2022b; Sun et al., 2023) can find new indications from existing drugs and expand their scopes and uses. Drug repositioning demonstrates several advantages compared to an entirely new drug design. First, it has less risk related to subsequent efficacy trial failure. Second, drug research and development time can be shortened because most of the preclinical testing and safety assessment have already been done. Finally, less investment is required because drug repositioning can still provide significant data in preclinical, clinical phase I, and clinical phase II stages. In summary, drug repositioning has been widely applied to DTI inference (Tian et al., 2022a; Zhang et al., 2022a).

To date, various drug repositioning methods (Chen et al., 2012, 2016) have been used to identify potential DTIs. These methods can be roughly divided into three categories: docking simulation (Guo et al., 2022; Peng et al., 2022a; Zhao et al., 2023), network-based methods, and machine learning-based methods. Docking simulation first obtains 3D structures of drugs and proteins and then runs molecular simulations to compute the binding ability for each drug-target pair (Li et al., 2006; Pujadas et al., 2008). However, 3D structures of a few proteins are unknown (for example, membrane proteins), thus it is not possible to detect potential drugs interacting with these proteins (Opella, 2013). Furthermore, docking simulation-based DTI identification can be challenging.

Network-based methods (Lotfi Shahreza et al., 2018) provide an efficient way for DTI prediction. Network-based methods integrate protein-protein similarity, drug-drug similarity, and known DTIs in to a heterogeneous network and develop network algorithms to find new DTIs (Chen et al., 2012). For example, Chen et al. (2012) proposed a random walk with a restart-based method. Mei et al. (2013) developed a bipartite local model BLM-NII. Van Laarhoven and Marchiori (2013) designed a computational model WNN-GIP by combining weighted nearest neighbor with Gaussian interaction profiles.

Machine learning-based methods use machine learning models to capture relationships between drugs and targets. For example, Precup et al. (2012); Buza and Peška (2017) designed K-nearest neighbor models with hubness-aware regression technique to alleviate the detrimental effect of bad hubs in a DTI network. In particular, deep learning has obtained wide application in DTI prediction. For example, Zong et al. (2017) developed a deep learning model based on the topology of a multipartite DTI network, Wang et al. (2018) used a deep ensemble learning model with a stacked autoencoder, Öztürk et al. (2018) designed a deep learning model with character representations, You et al. (2019) exploited a deep ensemble learning method with LASSO regression, Cheng et al. (2021) combined multi-head self-attention and graph attention network, Lee and Nam (2022) explored a sequence-based approach, Li et al. (2022) designed a dual-stream graph neural network, Mukherjee et al. (2022) used a deep graph convolutional network and LSTM, Zhang et al. (2022b) exploited a graph neural network, and Tayebi et al. (2022) designed a deep ensemble-balanced learning model.

The above three types of methods effectively identify potential DTIs. Network-based methods predict possible DTIs by combining topological information and node features in a DTI network. However, network-based methods cannot identify potential DTIs for new drugs or targets. Machine learning-based methods utilized feature information involved in drugs and targets and can significantly improve DTI prediction performance. However, machine learning-based methods are susceptive to data quality and feature selection and need huge amounts of data. In this study, we developed a novel DTI prediction method called EnGDD by combining initial feature acquisition, dimensional reduction, and DTI classification based on Gradient boosting neural network (Grownet) (Badirli et al., 2020), Deep Neural Network (DNN) and Deep Forest (DeepForest) (Zhou and Feng, 2019).

Parkinson's Disease (PD) and Alzheimer's Disease (AD) are two common neurodegenerative diseases. PD is mainly characterized by movement disorders, muscle stiffness, tremor, and other symptoms. Its pathogenesis involves many aspects including environment, genetics, and neurochemistry (Poewe et al., 2017). AD has cognitive impairment, memory loss, language impairment, and other symptoms. Its pathogenesis is still unclear. Although a few drugs have been applied to their therapies, new therapeutic clues are still essential to the two diseases (Iraji et al., 2020; Yiannopoulou and Papageorgiou, 2020; Liang et al., 2022b; Lin et al., 2022). Thus, we used the proposed EnGDD method and found new therapeutic clues for PD and AD.

## 2. Materials and methods

### 2.1. Data preparation

Yamanishi_08 has been widely used as a gold standard dataset in the field of DTI prediction. It was collected from the KEGG BRITE (Kanehisa et al., 2017), BRENDA (Schomburg et al., 2004), SuperTarget (Günther et al., 2007), and DrugBank (Wishart et al., 2008) databases. It was categorized into four DTI datasets based on different target proteins, that is, nuclear receptors (NR), G protein-coupled receptors (GPCR), ion channels (IC), and enzymes (E). Chu et al. (2021) collected new drugs, new targets, and new DTIs and further updated the four DTI datasets. On the four datasets, there are 886 DTIs between 541 drugs and 33 targets, 5383 DTIs between 1680 drugs and 156 targets, 6385 DTIs between 765 drugs and 238 targets, and 7371 DTIs between 1777 drugs and 1411 targets after an update, respectively. We used the four datasets to capture potential DTIs.

### 2.2. Initial feature acquisition

ChemDes (Dong et al., 2015) and BioTriangle (Dong et al., 2016) were used to extract the initial features of drugs and targets. ChemDes (Dong et al., 2015) is a freely available tool for molecular description and fingerprint calculation. A drug can be first represented by a Simplified Molecular Input Line Entry System (SMILES) string. SMILES string is then converted into fingerprints via ChemDes. In this study, we use ChemDes and describe each drug as a 1,538-dimensional vector.

BioTriangle (Dong et al., 2016) provides 14 types of biological features to represent each target protein. These features include amino acid composition, dipeptide composition, tripeptide composition, CTD composition, CTD transition, CTD distribution, M-B autocorrelation, Moran autocorrelation, Geary autocorrelation, conjoint triad features, quasi-sequence order descriptors, sequence order coupling number, pseudo amino acid composition 1, and pseudo amino acid composition 2. In this study, we use BioTriangle and describe each target as a 10029-dimensional vector.

### 2.3. Dimensional reduction

The dimensions of the extracted drug and target features are high and there is a large amount of robust information. We reduce the feature dimensions using Principal Component Analysis (PCA). PCA is a common machine learning algorithm mainly used for dimensionality reduction and feature extraction. It can map raw high-dimensional data into low-dimensional space while preserving main information and structure of data, thus making the prediction model more efficient and accurate. Finally, drugs and targets can be denoted as two *d*-dimensional vectors. In addition the two vectors are concatenated and each drug-target pair is represented using a 2*d*-dimensional vector *x*.

### 2.4. DTI classification

**Figure 1:**
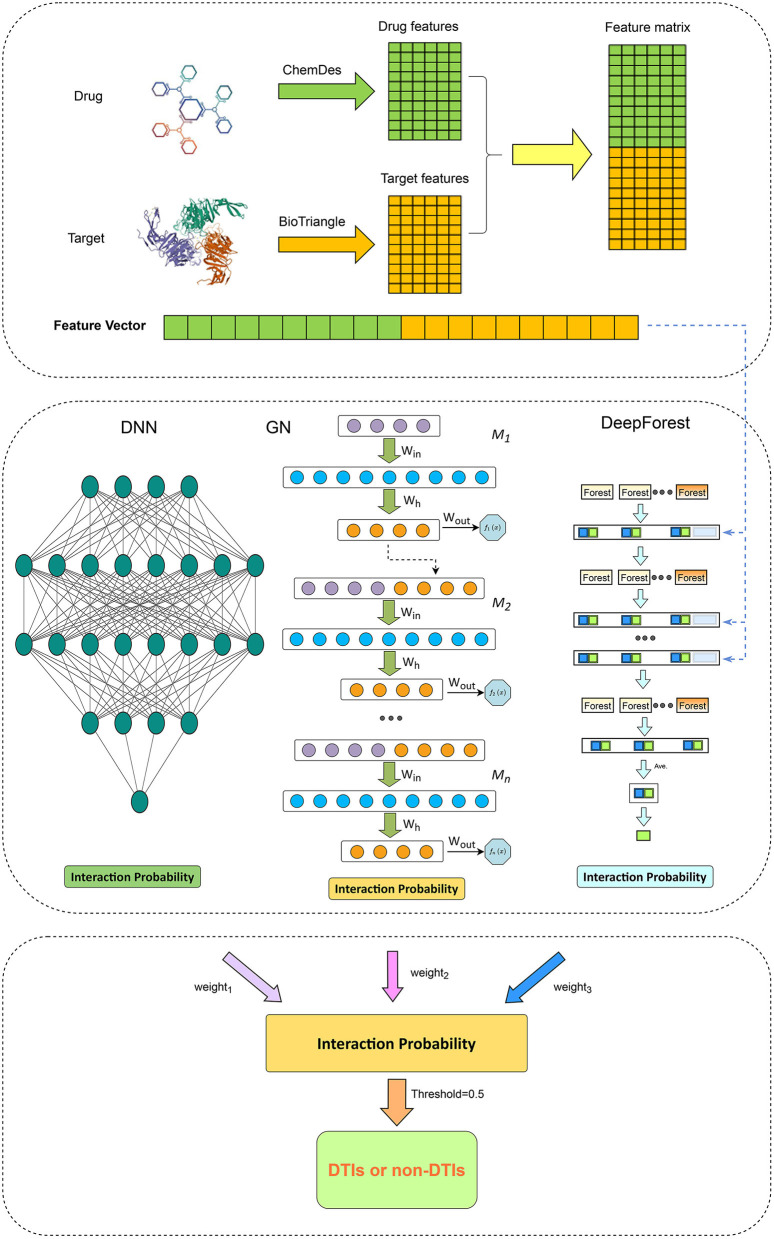
The pipeline for drug-target interaction prediction based on Grownet, DNN, and DeepForest.

We first compute interaction probability for each drug-target pair using Grownet, DNN, and DeepForest, respectively. The probability is then integrated by the soft voting and each drug-target can be classified. The details are shown in Figure 1.

#### 2.4.1. Gradient boosting neural network

Gradient boosting machine (Friedman, 2001; ZhouZhou et al., 2021) is a function estimation approach based on numerical optimization and obtains wide application (Peng et al., 2021). Grownet (Badirli et al., 2020) is a gradient boosting framework with shallow neural networks. As shown in Figure 1, Grownet uses shallow neural networks as basic learners and propagates information from the previous classifier to the next one.

At each layer, the learner is trained based on DTI features. The final output is one weighted sum of outputs from all learners: $\overset{k=K}{\underset{k=1}{\sum }}\alpha_{k}f_{k}\left(x\right)$ where *K* denotes the number of learners.

For a drug-target pair *x*_*i*_ with a 2*d*-dimensional feature in a DTI dataset $D=\left\{{\left(x_{i},y_{i}\right)}_{i=1}^{n}|x_{i}\in {\mathbb{R}}^{2d},y_{i}\in \mathbb{R}\right\}$, Grownet obtains its label through *K* additive functions by Equation (1):


(1) $$\begin{array}{l} {ŷ}_{i}=F\left(x_{i}\right)=\overset{K}{\underset{k=0}{\sum }}\alpha_{k}f_{k}\left(x_{i}\right),f_{k}\in \;F \end{array}$$

where F and α_*k*_ denote the space of multilayer perceptrons and the step size, respectively. *f*_*k*_ is a shallow neural network with linear activation function in the output layer.

Let ${ŷ}_{i}^{\left(t-1\right)}=\overset{t-1}{\underset{k=0}{\sum }}\alpha_{k}f_{k}\left(x_{i}\right)$ denote the output at the (*t*−1)th layer for *x*_*i*_, we minimize the following loss function by Equation (2):


(2) $${ℒ}^{\left(t\right)}=\sum_{i=0}^{n}l\left(y_{i},{\hat{y}}_{i}^{\left(t-1\right)}+\alpha_{t}f_{t}\left(x_{i}\right)\right)$$

Thus, the objective function is simplified by Equation (3):


(3) $$\begin{array}{l} L^{\left(t\right)}=\overset{n}{\underset{i=0}{\sum }}h_{i}{\left({ŷ}_{i}-\alpha_{t}f_{t}\left(x_{i}\right)\right)}^{2} \end{array}$$

where ŷ_*i*_ = −*g*_*i*_/*h*_*i*_, *g*_*i*_ and *h*_*i*_ denote the first-order and second-order gradients of the objective function at *x*_*i*_, respectively.

#### 2.4.2. Deep neural network

Deep neural networks have been broadly applied in the field of bioinformatics. In this section, we designed a DNN to classify unknown drug-target pairs. The constructed DNN comprises an input layer, multiple hidden layers, and a larger output layer. For a drug-target pair *x*_*i*_ with 2*d*-dimensional features, the input layer feeds *x*_*i*_ to the network.

We minimize the following binary cross-entropy function to quantify how many of the predicted labels differ from the real ones by Equation (4):


(4) $$\begin{array}{l} \begin{array}{c} L=-\frac{1}{n}\overset{n}{\underset{i=1}{\sum }}\left[y_{i}\log{\hat{y}}_{i}+\left(1-y_{i}\right)\log\left(1-{\hat{y}}_{i}\right)\right]. \end{array} \end{array}$$

where ***y***_*i*_ and ${\hat{y}}_{i}$ denote true labels and the predicted interaction probability of *x*_*i*_. We use the Adam algorithm (Kingma and Ba, 2014) to train the DNN. The training is implemented with 100 epochs and each epoch has a mini-batch with the size of 64.

The final output layer with a single neuron and the sigmoid function is used to output an interaction probability for *x*_*i*_ by Equation (5):


(5) $$\begin{array}{l} y_{i}=\frac{1}{1+e^{-x_{i}}} \end{array}$$

#### 2.4.3. Deep forest

To solve complex tasks, learning models increasingly go deep (Cai et al., 2021; Li et al., 2022). Non-neural network style-based deep models demonstrate powerful learning abilities when they can go deep. DeepForest (Zhou and Feng, 2019) is a non-neural network style deep learning model and is constructed upon multi-grained cascade framework. It demonstrates the powerful classification performance and less training time.

In this study, we used DeepForest with no more than 20 layers to classify unobserved drug-target pairs. We choose random forests (Qi, 2012; Biau and Scornet, 2016) and extra trees (Geurts et al., 2006) as basic classifiers in DeepForest. Random forest (Qi, 2012; Biau and Scornet, 2016) is a non-parametric and interpretable classification model. It is an ensemble of many random decision trees and has a better performance in classification tasks with complex data structure, small sample size, and high-dimensional feature space. An extra tree (Geurts et al., 2006) is an ensemble of unpruned decision trees. It can better reduce variance by completely randomly selecting cut-points and minimizing classification bias by using whole learning samples.

As shown in Figure 1, each cascade layer in DeepForest comprises five random forests and five extra trees. Each predictor consists of 100 decision trees. In each layer, each predictor computes a ratio of a given DTI feature belonging to a positive or negative class. The predicted probabilities from all learners produce a class vector. The vector in addition to the raw DTI feature vector is used as input in the next layer.

In particular, similar to DNN, deep forest utilizes a cascade structure. In the structure, each level receives features from its preceding level, and outputs the results to the next level. Therefore, although the proportion of a 20-dimensional class vector in the input layer may be relatively smaller, its proportion in a DTI feature vector will increase with the deepening of the number of layers. Therefore, the 20-dimensional class vector cannot be drowned out in DeepForest.

#### 2.4.4. Ensemble learning

Ensemble learning demonstrates better classification performance than a single classifier. Thus, we combined Grownet, DNN, and DeepForest and developed a hybrid model for DTI identification based on the soft voting approach by Equation (6):


(6) $$\begin{array}{l} Score=\alpha C_{Grownet}+\beta C_{DNN}+\gamma C_{DeepForest} \end{array}$$

where *C*_*Grownet*_, *C*_*DNN*_, and *C*_*deepforest*_ represent DTI prediction results from Grownet, DNN, and DeepForest, respectively. α, β, and γ denote the corresponding weights. In particular, one drug-target pair is labeled as positive if its interaction score is greater than 0.5; otherwise, the pair is classified as negative.

## 3. Results

### 3.1. Evaluation metrics

In the experiments, precision, recall, accuracy, F1 score, AUC, and AUPR were used to measure the classification performance of our proposed EnGDD method. Higher values indicate better prediction ability for the above metrics. The experiments were repeated 20 times and their average values were selected as the final results. The former four metrics are defined by Equations (7)–(10):


(7) $$\begin{array}{l} Precision=\frac{TP}{TP+FP} \end{array}$$

(8) $$\begin{array}{l} Recall=\frac{TP}{TP+FN} \end{array}$$

(9) $$\begin{array}{l} Accuracy=\frac{TP+TN}{TP+TN+FP+FN} \end{array}$$

(10) $$\begin{array}{l} F1-Score=\frac{2\cdot Precision\cdot Recall}{Precision+Recall} \end{array}$$

where TP, FP, FN, and TN indicate true positives, false positives, false negatives, and true negatives.

AUC denotes the area under the receiver operating characteristic (ROC) curve and AUPR denotes the area under the Precision-Recall (PR) curve.

### 3.2. Experimental settings

PaDEL in ChemDes was used to extract drug features. The number of drug features obtained from PaDEL were as follows: 120 constitutional descriptors, 346 autocorrelation descriptors, 42 basak descriptors, 6 BCUT descriptors, 96 burden descriptors, 56 connectivity descriptors, 489 E-state descriptors, 3 Kappa descriptors, 15 molecular property descriptors, 6 quantum chemical descriptors, and 265 topological descriptors. All features in BioTriangle were applied to depict target proteins. Finally, we obtained one 100-dimensional feature for drugs and targets after dimensional reduction, respectively.

In addition, to obtain more accurate and stable prediction results, we used grid search to set the final parameters in the ensemble model. Grid search is a common hyperparameter optimization method and can be used to determine the final parameters in a model. We used it to traverse the parameter space and try all possible hyperparameter combinations. The optimal parameter combination is then selected as the final parameters of the ensemble model. Experimental settings in DNN were the same as Zhou et al. (2021). For Deepforest, we set max_layers = 20, n_estimators = 5, n_trees = 100, predictor = “forest”, and max_depth = None. For Grownet, we set lr = 0.05, num_nets = 20, batch_size = 64, boost_rate = 1.0, epochs_per_stage = 1, correct_epoch = 1, and L2 = 0.001.

Three 5-fold cross validations (CVs) were performed to assess the DTI prediction performance of EnGDD:

1. Five-fold CV on drugs (*CV*_*d*_, DTI prediction for new drugs): 80% of drugs were randomly selected as training data and the remaining 20% was taken as test data in each round.
2. Five-fold CV on targets (*CV*_*t*_, DTI prediction for new targets): 80% of targets were randomly selected as training data and the remaining 20% is taken as test data in each round.
3. Five-fold CV on drug-target pairs (*CV*_*dt*_, DTI prediction for drug-target pairs): 80% of drug-target pairs were randomly selected as training data and the remaining 20% is taken as test data in each round.

There are a few positive DTIs and it is a lack of negative DTIs on the four DTI datasets. If negative DTIs are not reasonably selected, it is easy to cause overfitting. Undersampling is an approach that deals with the data imbalance problem, and has been used to address situations where the number of samples in one category of data is far less than that in the other categories. Consequently, we used an undersampling approach to balance the datasets. That is, the ratio of positive drug-target pairs to negative drug-target pairs is set to 1 to solve the data imbalance problem.

### 3.3. Comparison with seven state-of-the-art DTI prediction methods

We compared the proposed EnGDD algorithm with seven state-of-the-art DTI prediction models to measure the classification ability of EnGDD, i.e., BLM-NII, NRLMF, WNNGIP, NEDTP, DTi2Vec, RoFDT, and MolTrans. To identify potential DTIs, BLM-NII (Mei et al., 2013) used a bipartite local model and neighbor interaction profiles, NRLMF (Liu et al., 2016) designed a neighborhood regularized logistic matrix factorization method, WNNGIP (Van Laarhoven and Marchiori, 2013) combined a weighted nearest neighbor profile and Gaussian interaction profile, NEDTP (An and Yu, 2021) is a heterogeneous network embedding framework, DTi2Vec (Thafar et al., 2021) integrated network embedding and ensemble learning, RoFDT (Wang et al., 2022) proposed a rotation forest model. Huang et al. (2021) proposed a molecular interaction transformer (MolTrans) to capture possible DTIs. MolTrans first designed an augmented transformer encoder to extract the semantic relationships among sub-structures from unlabeled biomedical data and then used a knowledge inspired sub-structural pattern detection method for more accurate DTI prediction.

**Figure 2:**
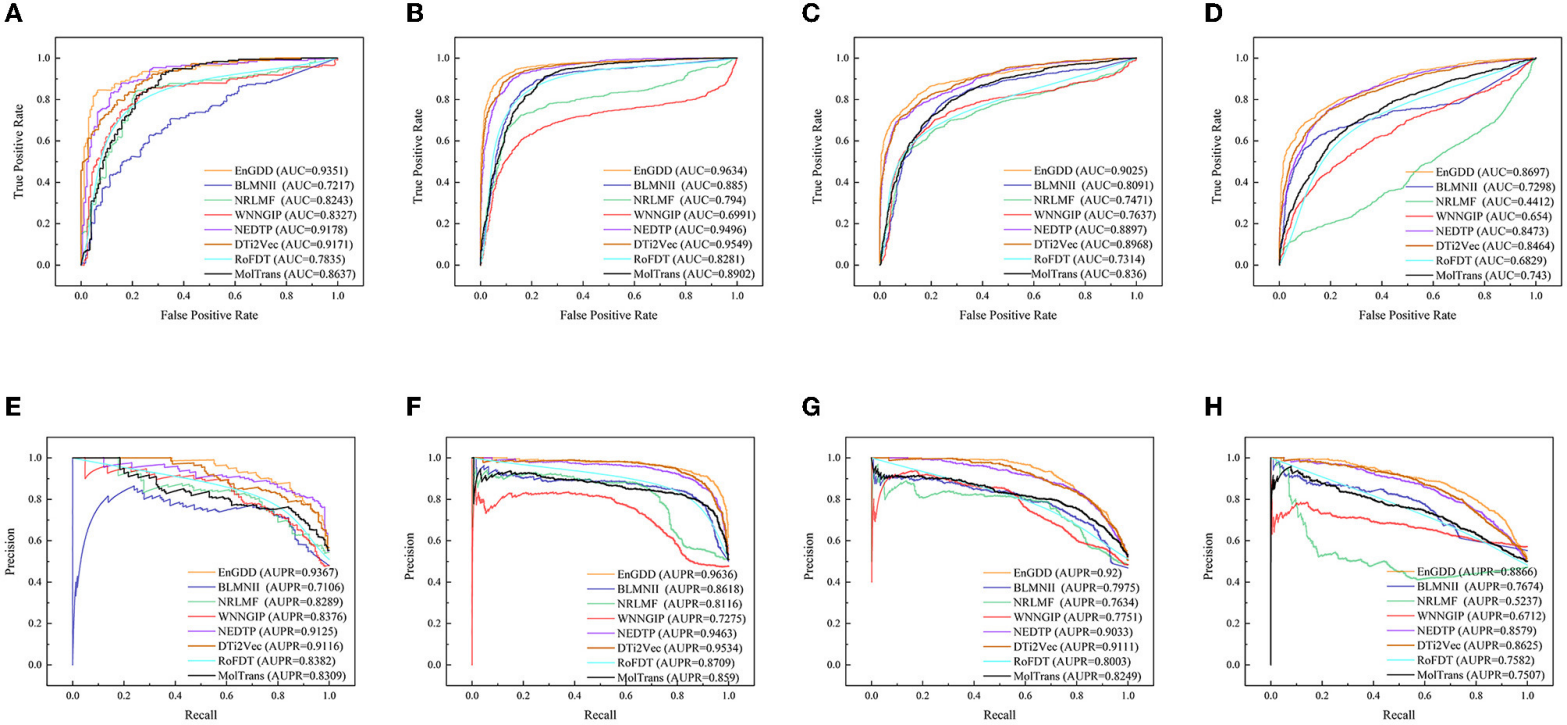
The ROC and PR curves of eight models under *CV*_*d*_. Subfigures **(A–D)** denote the ROC curves of all methods on the nuclear receptor, GPCR, ion channel, and enzyme datasets. Subfigures **(E–H)** denote the precision-recall curves of all methods on the nuclear receptor, GPCR, ion channel, and enzyme datasets under *CV*_*d*_.

Table 1 illustrates the DTI prediction performance of EnGDD and the other seven DTI prediction models under *CV*_*d*_. From Table 1, we observed that although EnGDD computed smaller precisions on GPCRs and ion channels than MolTrans. It computed better accuracy, F1-score, AUC, and AUPR than the other seven DTI prediction models. In particular, EnGDD obtained the best AUCs and AUPRs on nuclear receptors, GPCR, ion channels, and enzymes among the eight DTI prediction models. It computed AUCs of 0.9351, 0.9634, 0.9025, and 0.8697 on the four datasets, outperforming 1.85%, 0.88%, 0.63%, and 2.58% than the second-best approach, respectively. It calculated AUPRs of 0.9367, 0.9636, 0.9200, and 0.8855 on the four datasets, better 2.58%, 1.06%, 0.97%, and 2.60% than the second-best approach, respectively. Figure 2 shows the ROC and PR curves of the eight DTI prediction models and corresponding AUCs and AUPRs on four DTI datasets. The above results demonstrate that EnGDD obtained powerful DTI prediction performance under *CV*_*d*_ and can be applied to effectively find potential targets for new drugs.

**Table 1:** Performance of eight DTI prediction methods on *CV*_*d*_.

**Metric**	**Dataset**	**EnGDD**	**BLM-NII**	**NRLMF**	**WNNGIP**	**NEDTP**	**DTi2Vec**	**RoFDT**	**MolTrans**
Precision	NR	**0.8309**	0.6959	0.7285	0.7336	0.8193	0.8198	0.7888	0.7590
		GPCR	0.8686	0.8219	0.7090	0.6605	0.8808	**0.8938**	0.8278	0.7748
		IC	0.8400	0.7640	0.6821	0.6840	0.8643	**0.8874**	0.7763	0.7314
		E	0.7917	0.7675	0.4967	0.6212	0.8508	**0.8599**	0.7121	0.6289
Recall	NR	**0.9057**	0.5327	0.6973	0.7143	0.8795	0.8709	0.7750	0.8925
		GPCR	**0.9422**	0.6746	0.7032	0.6384	0.8966	0.8905	0.8280	0.9132
		IC	0.8142	0.5952	0.6705	0.6937	0.7330	0.7283	0.6488	**0.8387**
		E	0.7789	0.5082	0.4780	0.6055	0.6168	0.6147	0.6089	**0.8002**
Accuracy	NR	**0.8601**	0.6408	0.6817	0.6909	0.8425	0.8395	0.7834	0.8026
		GPCR	**0.8998**	0.7471	0.6552	0.6068	0.8876	0.8923	0.8280	0.8236
		IC	**0.8296**	0.6758	0.6266	0.6486	0.8091	0.8180	0.7313	0.7627
		E	**0.7869**	0.6528	0.4681	0.5807	0.7546	0.7575	0.6824	0.6615
F1-score	NR	**0.8663**	0.5992	0.7123	0.7235	0.8479	0.8442	0.7812	0.8193
		GPCR	**0.9039**	0.7407	0.7060	0.6489	0.8885	0.8921	0.8278	0.8382
		IC	**0.8264**	0.6684	0.6762	0.6885	0.7928	0.7993	0.7059	0.7804
		E	**0.7848**	0.6070	0.4867	0.6128	0.7140	0.7160	0.6554	0.7035
AUC	NR	**0.9351**	0.7217	0.8243	0.8327	0.9178	0.9171	0.7835	0.8637
		GPCR	**0.9634**	0.8850	0.7940	0.6991	0.9496	0.9549	0.8281	0.8902
		IC	**0.9025**	0.8091	0.7471	0.7637	0.8897	0.8968	0.7314	0.8360
		E	**0.8697**	0.7298	0.4412	0.6540	0.8473	0.8464	0.6829	0.7430
AUPR	NR	**0.9367**	0.7106	0.8289	0.8376	0.9125	0.9116	0.8382	0.8309
		GPCR	**0.9636**	0.8618	0.8116	0.7275	0.9463	0.9534	0.8709	0.8590
		IC	**0.9200**	0.7975	0.7634	0.7751	0.9033	0.9111	0.8003	0.8249
		E	**0.8855**	0.7678	0.5230	0.6720	0.8579	0.8625	0.7582	0.7507

The bold value denotes the best performance in each row.

**Figure 3:**
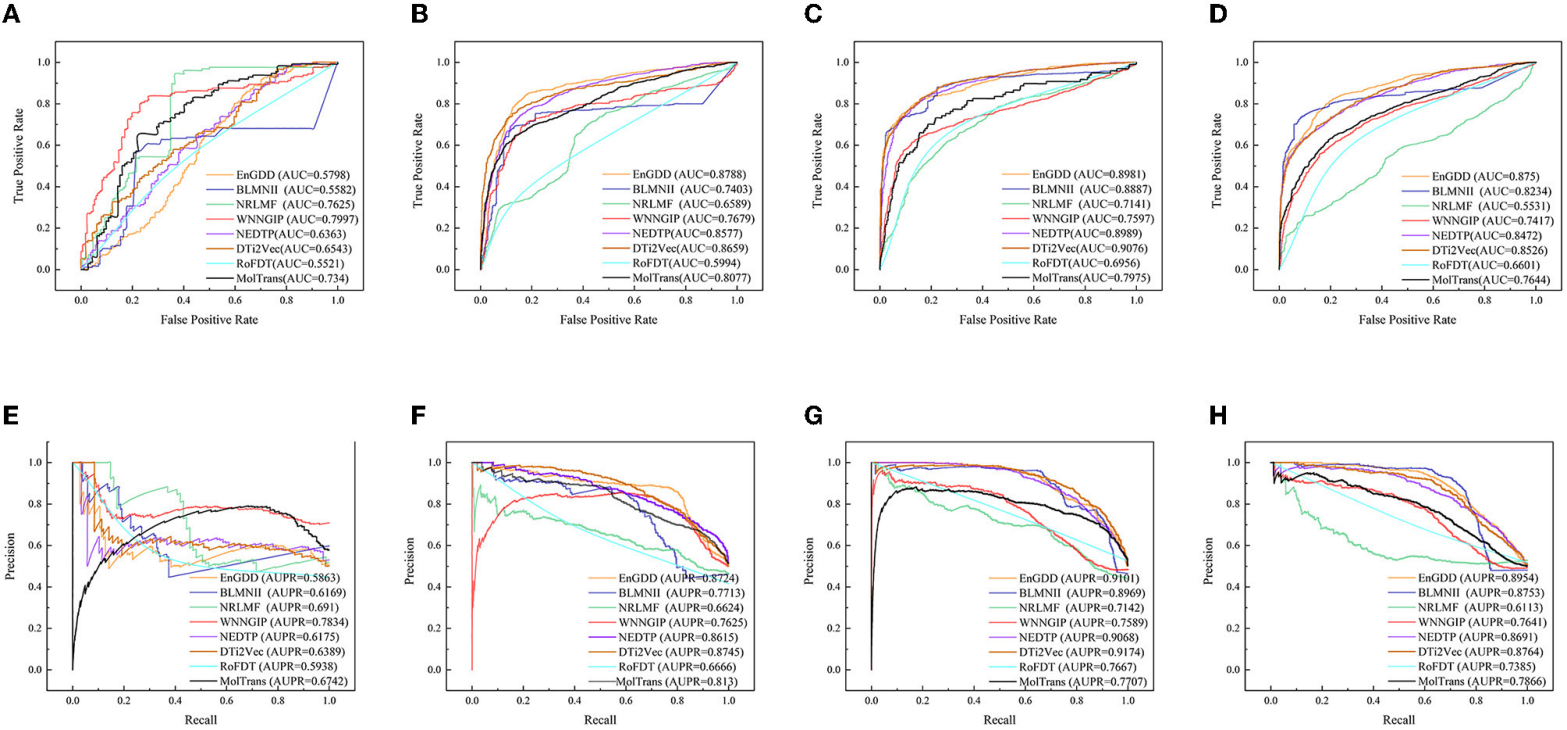
The ROC and PR curves of eight models under *CV*_*t*_. Subfigures **(A–D)** denote the ROC curves of all methods on the nuclear receptor, GPCR, ion channel, and enzyme datasets. Subfigures **(E–H)** denote the precision-recall curves of all methods on the nuclear receptor, GPCR, ion channel, and enzyme datasets under *CV*_*t*_.

Table 2 shows the DTI inference performance of the eight DTI prediction models under *CV*_*t*_. The results from Table 2 shows that EnGDD computed lower performance on nuclear receptors than WNNGIP. It may be caused by a small sample size on nuclear receptors. It significantly outperformed WNNGIP on GPCRs, ion channels, and enzymes, which contain larger samples. In addition, although DTi2Vec computed better precision, AUC, and AUPR than EnGDD on ion channels, the differences are very small. It may be caused by its different data structure. Figure 3 gives the ROC and PR curves of the eight models and corresponding AUCs and AUPRs on the four DTI datasets. In summary, EnGDD can be used to screen potential drugs for new targets.

**Table 2:** Performance of eight DTI prediction methods on *CV*_*t*_.

**Metric**	**Dataset**	**EnGDD**	**BLM-NII**	**NRLMF**	**WNNGIP**	**NEDTP**	**DTi2Vec**	**RoFDT**	**MolTrans**
Precision	NR	0.6357	0.5926	0.6543	**0.6994**	0.6350	0.6592	0.5270	0.6481
		GPCR	0.8700	0.7324	0.6186	0.6834	0.8852	**0.9054**	0.6407	0.6863
		IC	0.8542	0.7832	0.6589	0.6844	0.8954	**0.9122**	0.7274	0.6890
		E	0.8101	0.8487	0.5696	0.6759	0.8709	**0.8770**	0.6923	0.6366
Recall	NR	0.2285	0.4223	0.5502	0.6549	0.2570	0.2774	0.3453	**0.8787**
		GPCR	0.6670	0.5579	0.5982	0.6794	0.5571	0.5797	0.3999	**0.8498**
		IC	0.7937	0.7310	0.6284	0.6730	0.6995	0.7094	0.6134	**0.8681**
		E	0.7892	0.6222	0.5479	0.6745	0.6370	0.6578	0.5708	**0.8028**
Accuracy	NR	0.5440	0.5358	0.6412	0.6706	0.5579	0.5679	0.5552	**0.6974**
		GPCR	**0.7836**	0.6316	0.5823	0.6531	0.7421	0.7593	0.6043	0.7222
		IC	**0.8286**	0.7225	0.6144	0.6499	0.8088	0.8205	0.6957	0.7300
		E	**0.8020**	0.7256	0.5263	0.6320	0.7714	0.7829	0.6604	0.6663
F1-score	NR	0.3226	0.4715	0.5954	0.6734	0.3508	0.3780	0.3810	**0.7440**
		GPCR	0.7498	0.6312	0.6065	0.6799	0.6740	0.7000	0.4839	**0.7542**
		IC	**0.8215**	0.7555	0.6427	0.6782	0.7824	0.7958	0.6624	0.7650
		E	**0.7992**	0.7175	0.5583	0.6746	0.7350	0.7509	0.6249	0.7068
AUC	NR	0.5798	0.5582	0.7625	**0.7997**	0.6363	0.6543	0.5521	0.7340
		GPCR	**0.8788**	0.7403	0.6589	0.7679	0.8577	0.8659	0.5994	0.8077
		IC	0.8981	0.8887	0.7141	0.7597	0.8989	**0.9076**	0.6956	0.7975
		E	**0.8750**	0.8234	0.5531	0.7417	0.8472	0.8526	0.6601	0.7644
AUPR	NR	0.5863	0.6169	0.6910	**0.7834**	0.6175	0.6389	0.5938	0.6742
		GPCR	0.8724	0.7713	0.6624	0.7625	0.8615	**0.8745**	0.6666	0.8130
		IC	0.9101	0.8969	0.7142	0.7589	0.9068	**0.9174**	0.7667	0.7707
		E	**0.8954**	0.8753	0.6113	0.7641	0.8691	0.8764	0.7385	0.7866

The bold value denotes the best performance in each row.

**Figure 4:**
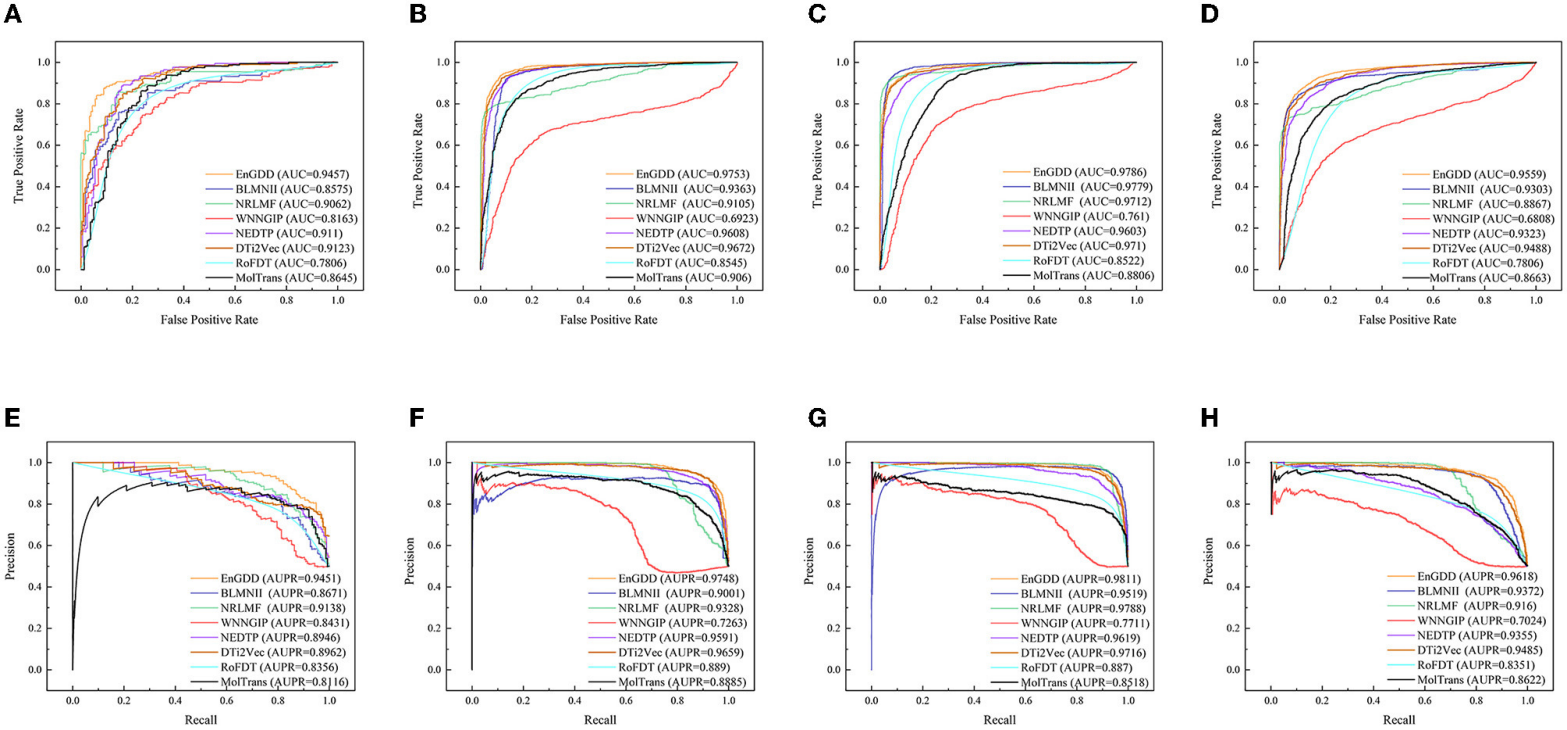
The ROC and PR curves of eight models under *CV*_*dt*_. Subfigures **(A–D)** denote the ROC curves of all methods on the nuclear receptor, GPCR, ion channel, and enzyme datasets. Subfigures **(E–H)** denote the precision-recall curves of all methods on the nuclear receptor, GPCR, ion channel, and enzyme datasets under *CV*_*dt*_.

Table 3 gives DTI prediction results of the eight models under *CV*_*dt*_. The results from Table 3 demonstrate that although EnGDD computed sightly lower precisions, accuracies, and F1-scores than DTi2Vec, it obtained the best recalls, AUCs, and AUPRs on the four DTI datasets. AUC and AUPR are two more important evaluation metrics. EnGDD computed the best AUCs of 0.9457, 0.9753, 0.9786, and 0.9559, outperforming 3.53%, 0.83%, 0.07%, and 1.16% than the second-best approach on the four datasets, respectively. It achieved the best AUPRs of 0.9451, 0.9748, 0.9811, and 0.9618, better 3.31%, 0.91%, 0.23%, and 1.38% than the second-best approach, respectively. Figure 4 shows the ROC and PR curves of the eight models and corresponding AUCs and AUPRs on the datasets. The results suggest that EnGDD can better capture possible DTIs from unknown drug-target pairs.

**Table 3:** Performance of eight DTI prediction methods on *CV*_*dt*_.

**Metric**	**Dataset**	**EnGDD**	**BLM-NII**	**NRLMF**	**WNNGIP**	**NEDTP**	**DTi2Vec**	**RoFDT**	**MolTrans**
Precision	NR	**0.8286**	0.7487	0.7885	0.7223	0.8157	0.8160	0.7772	0.7620
		GPCR	0.8699	0.8518	0.7625	0.6591	0.8840	**0.8980**	0.8401	0.8091
		IC	0.8638	0.8707	0.8207	0.6949	0.8823	**0.9051**	0.8351	0.7734
		E	0.8196	0.8308	0.7365	0.6414	0.8727	**0.8832**	0.7652	0.7867
Recall	NR	**0.9294**	0.7344	0.7692	0.7242	0.8814	0.8739	0.7878	0.9066
		GPCR	**0.9675**	0.7288	0.8500	0.6324	0.9214	0.9304	0.8758	0.8831
		IC	**0.9670**	0.8068	0.9228	0.6770	0.9121	0.9309	0.8777	0.8851
		E	**0.9430**	0.8208	0.8374	0.6259	0.8453	0.8788	0.8098	0.8142
Accuracy	NR	**0.8682**	0.7075	0.7527	0.6826	0.8406	0.8379	0.7806	0.8102
		GPCR	0.9113	0.7862	0.7600	0.6012	0.9002	**0.9123**	0.8545	0.8369
		IC	0.9072	0.8342	0.8360	0.6461	0.8951	**0.9166**	0.8522	0.8119
		E	0.8676	0.8003	0.7346	0.5954	0.8609	**0.8813**	0.7806	0.7965
F1-score	NR	**0.8758**	0.7407	0.7783	0.7229	0.8469	0.8436	0.7820	0.8270
		GPCR	**0.9161**	0.7853	0.8038	0.6452	0.9023	0.9139	0.8575	0.8440
		IC	0.9125	0.8373	0.8687	0.6856	0.8969	**0.9178**	0.8558	0.8249
		E	0.8769	0.8253	0.7837	0.6333	0.8587	**0.8810**	0.7868	0.7999
AUC	NR	**0.9457**	0.8575	0.9062	0.8163	0.9110	0.9123	0.7806	0.8645
		GPCR	**0.9753**	0.9363	0.9105	0.6923	0.9608	0.9672	0.8545	0.9060
		IC	**0.9786**	0.9779	0.9712	0.7610	0.9603	0.9710	0.8522	0.8806
		E	**0.9559**	0.9303	0.8867	0.6808	0.9323	0.9448	0.7806	0.8663
AUPR	NR	**0.9451**	0.8671	0.9138	0.8431	0.8946	0.8962	0.8356	0.8116
		GPCR	**0.9748**	0.9001	0.9328	0.7263	0.9591	0.9659	0.8890	0.8885
		IC	**0.9811**	0.9519	0.9788	0.7711	0.9619	0.9716	0.8870	0.8518
		E	**0.9618**	0.9372	0.9160	0.7024	0.9355	0.9485	0.8351	0.8622

The bold value denotes the best performance in each row.

### 3.4. Comparison of EnGDD with three individual models

Our proposed EnGDD method combined three individual deep learning models, that is, Grownet, DNN, and DeepForest. In order to measure the performance of ensemble learning on DTI prediction, we compared EnGDD with the three models under three different cross validations (*CV*_*d*_, *CV*_*t*_, and *CV*_*dt*_). Tables 4–6 show the comparison results under the three cross validations.

**Table 4:** Performance of EnGDD and three individual models on *CV*_*d*_.

**Metric**	**Dataset**	**Grownet**	**DNN**	**DeepForest**	**EnGDD**
Precision	NR	0.7822	**0.8636**	0.8343	0.8309
		GPCR	0.7298	0.8944	**0.9361**	0.8686
		IC	0.6610	0.8565	**0.9497**	0.8400
		E	0.6475	0.8120	**0.9189**	0.7917
Recall	NR	**0.9168**	0.8734	0.8609	0.9057
		GPCR	**0.9755**	0.8955	0.8215	0.9422
		IC	**0.9234**	0.7702	0.6080	0.8142
		E	**0.9123**	0.7039	0.5211	0.7789
Accuracy	NR	0.8302	**0.8674**	0.8446	0.8601
		GPCR	0.8067	0.8949	0.8826	**0.8998**
		IC	0.7244	0.8206	0.7878	**0.8296**
		E	0.7074	0.7706	0.7377	**0.7869**
F1-score	NR	0.8439	**0.8680**	0.8468	0.8663
		GPCR	0.8347	0.8948	0.8748	**0.9039**
		IC	0.7702	0.8106	0.7397	**0.8264**
		E	0.7573	0.7535	0.6639	**0.7848**
AUC	NR	0.9045	0.9302	0.9263	**0.9351**
		GPCR	0.9455	0.9463	0.9595	**0.9634**
		IC	0.8781	0.8767	0.8847	**0.9025**
		E	0.8437	0.8391	0.8440	**0.8697**
AUPR	NR	0.8893	0.9189	0.9276	**0.9367**
		GPCR	0.9423	0.9369	0.9606	**0.9636**
		IC	0.8888	0.8901	0.9054	**0.9200**
		E	0.8546	0.8456	0.8655	**0.8855**

The bold value denotes the best performance in each row.

**Table 5:** Performance of EnGDD and three individual models on *CV*_*t*_.

**Metric**	**Dataset**	**Grownet**	**DNN**	**DeepForest**	**EnGDD**
Precision	NR	0.5292	0.5800	**0.7071**	0.6357
		GPCR	0.7004	0.7361	**0.9219**	0.8700
		IC	0.6756	0.8043	**0.9709**	0.8542
		E	0.6537	0.8076	**0.9403**	0.8101
Recall	NR	0.2888	**0.3014**	0.1227	0.2285
		GPCR	**0.8762**	0.3588	0.0714	0.6670
		IC	**0.9082**	0.6824	0.4991	0.7937
		E	**0.8958**	0.7173	0.5519	0.7892
Accuracy	NR	0.5141	**0.5652**	0.5378	0.5440
		GPCR	0.7492	0.6230	0.5333	**0.7836**
		IC	0.7360	0.7574	0.7422	**0.8286**
		E	0.7106	0.7734	0.7585	**0.8020**
F1-score	NR	**0.3693**	0.3607	0.2021	0.3226
		GPCR	**0.7767**	0.4564	0.1310	0.7498
		IC	0.7746	0.7351	0.6545	**0.8215**
		E	0.7557	0.7590	0.6949	**0.7992**
AUC	NR	0.5366	0.6456	**0.7004**	0.5798
		GPCR	0.8591	0.6991	0.8114	**0.8788**
		IC	0.8640	0.8048	0.8718	**0.8981**
		E	0.8432	0.8382	0.8500	**0.8750**
AUPR	NR	0.5363	0.6079	**0.6757**	0.5863
		GPCR	0.8701	0.6947	0.8005	**0.8724**
		IC	0.8729	0.8131	0.8957	**0.9101**
		E	0.8589	0.8463	0.8763	**0.8954**

The bold value denotes the best performance in each row.

**Table 6:** Performance of EnGDD and three individual models on *CV*_*dt*_.

**Metric**	**Dataset**	**Grownet**	**DNN**	**DeepForest**	**EnGDD**
Precision	NR	0.7817	**0.8594**	0.8314	0.8286
		GPCR	0.7528	0.8948	**0.9353**	0.8699
		IC	0.7056	0.8778	**0.9526**	0.8638
		E	0.6745	0.8467	**0.9210**	0.8196
Recall	NR	0.9289	0.8910	0.8858	**0.9294**
		GPCR	**0.9827**	0.9203	0.9184	0.9675
		IC	**0.9851**	0.9114	0.9267	0.9670
		E	**0.9695**	0.8779	0.8812	0.9430
Accuracy	NR	0.8345	**0.8723**	0.8526	0.8682
		GPCR	0.8298	0.9060	**0.9274**	0.9113
		IC	0.7868	0.8921	**0.9402**	0.9072
		E	0.7506	0.8593	**0.9028**	0.8676
F1-score	NR	0.8488	0.8745	0.8574	**0.8758**
		GPCR	0.8525	0.9073	**0.9267**	0.9161
		IC	0.8222	0.8941	**0.9394**	0.9125
		E	0.7955	0.8619	**0.9006**	0.8769
AUC	NR	0.9155	0.9327	0.9317	**0.9457**
		GPCR	0.9588	0.9551	**0.9770**	0.9753
		IC	0.9490	0.9478	**0.9820**	0.9786
		E	0.9200	0.9229	**0.9584**	0.9559
AUPR	NR	0.8976	0.9217	0.9286	**0.9451**
		GPCR	0.9554	0.9467	**0.9777**	0.9748
		IC	0.9458	0.9417	**0.9842**	0.9811
		E	0.9237	0.9182	**0.9640**	0.9618

The bold value denotes the best performance in each row.

As shown in Table 4, EnGDD obtained the best accuracy, F1-score, AUC, and AUPR on GPCR, ion channel, and enzyme under *CV*_*d*_. Although EnGDD computed slightly lower accuracy and F1 score than DNN on the nuclear receptor, the differences are only 0.0073 and 0.0017. Although EnGDD calculated relatively lower precision and recall than DeepForest and Grownet, respectively, it computed a better F1 score than them. The results show that EnGDD can be appropriate to screen possible targets for a new drug.

As shown in Table 5, EnGDD obtained better accuracy, AUC, and AUPR on GPCR, ion channel, and enzyme under *CV*_*t*_. In particular, all methods computed very low recall and F1 score and relatively low precision, accuracy, AUC, and AUPR on nuclear receptors under *CV*_*t*_. There are only 33 targets for nuclear receptor. When conducting cross validation on targets, the testing set only contains about 6 targets. Thus, all methods calculated lower performance on nuclear receptors.

As shown in Table 6, although EnGDD computed slightly lower performance than DeepForest under *CV*_*dt*_, the values are very tiny and can be neglected especially compared with the ones under *CV*_*d*_ and *CV*_*t*_. Thus, we used an ensemble of Grownet, DNN, and DeepForest to identify potential DTIs.

## 4. Case study

### 4.1. Possible DTI prediction from unknown drug-target pairs

**Figure 5:**
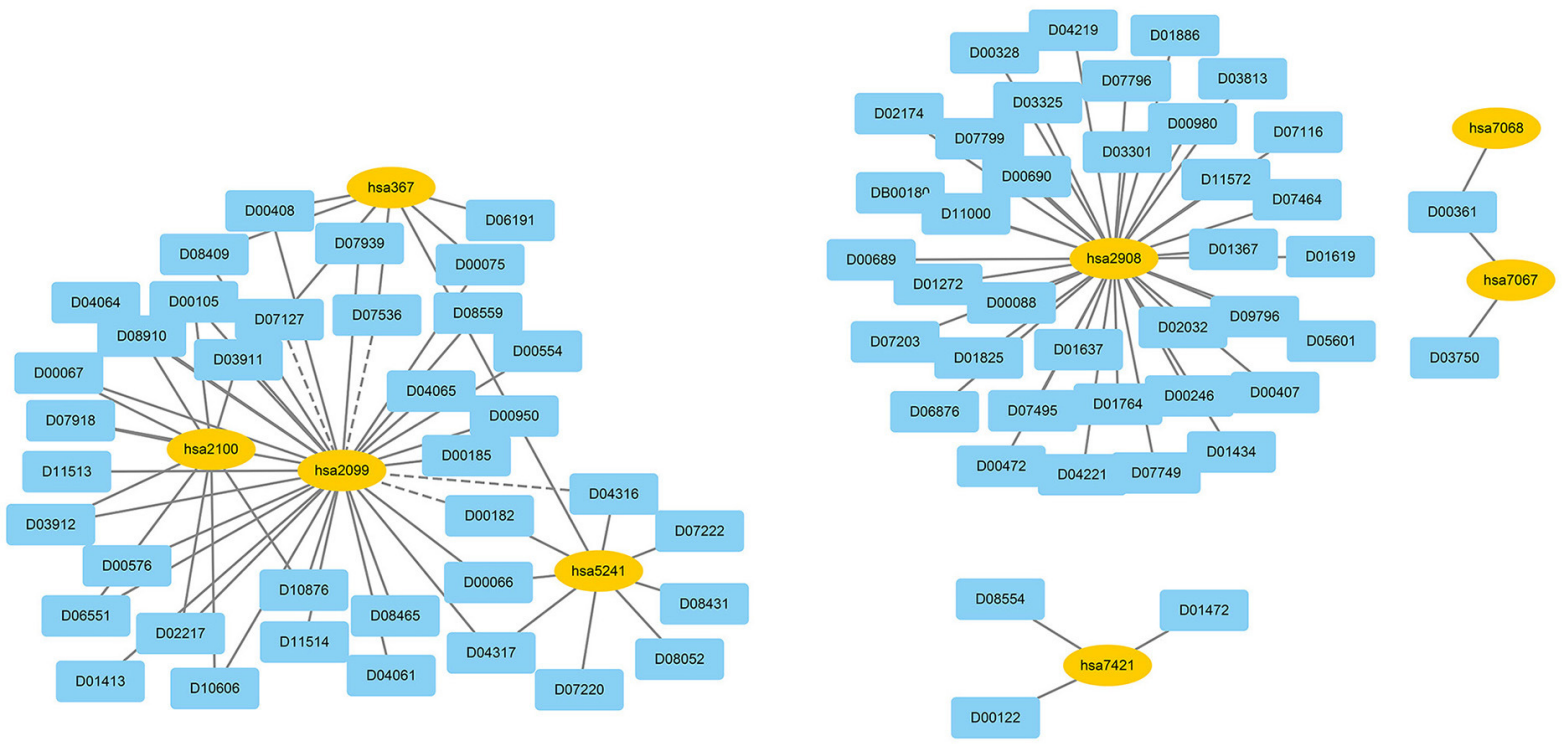
The predicted top 100 DTIs on nuclear receptors.

**Figure 6:**
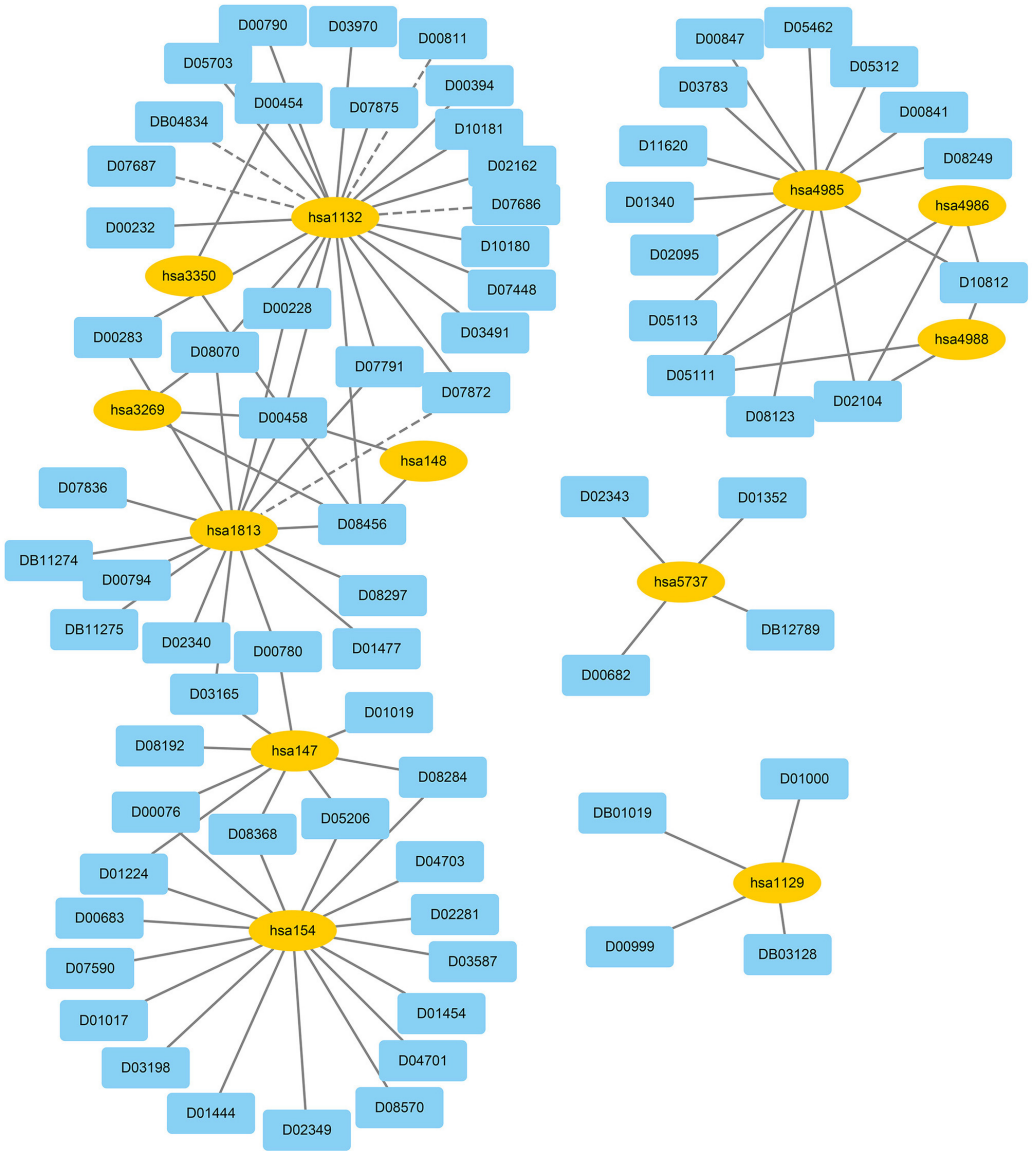
The predicted top 100 DTIs on GPCRs.

**Figure 7:**
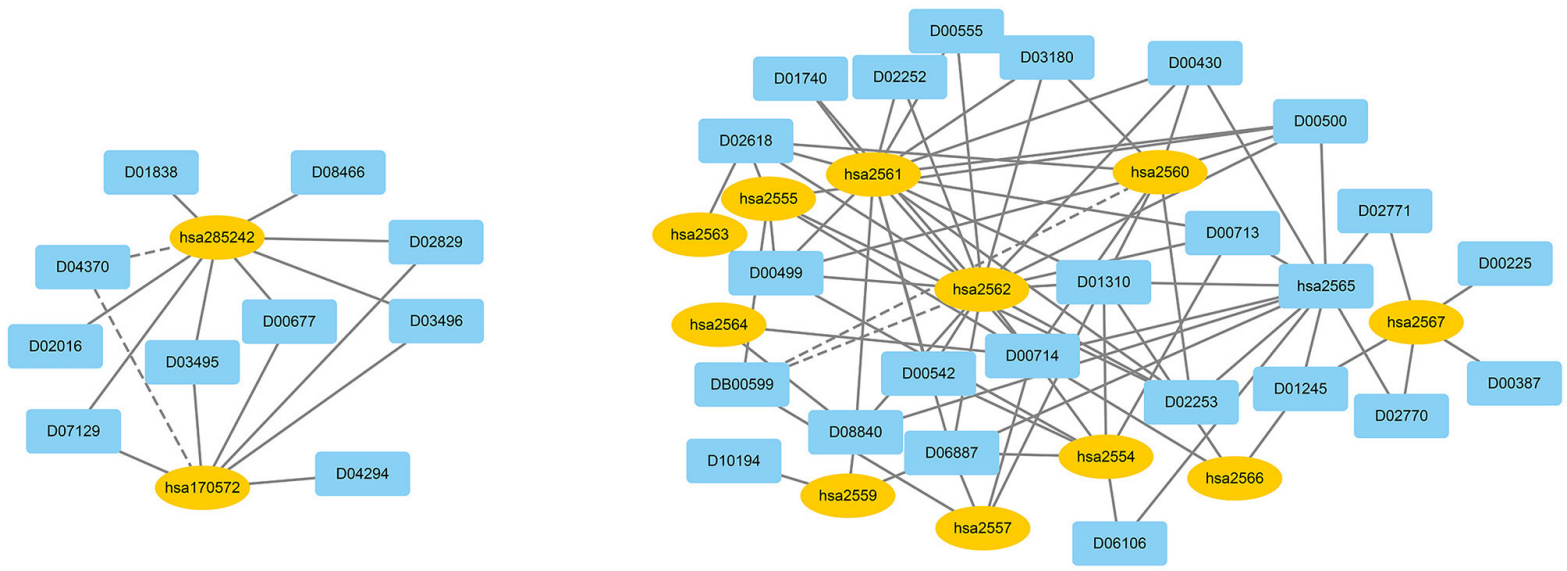
The predicted top 100 DTIs on ion channels.

**Figure 8:**
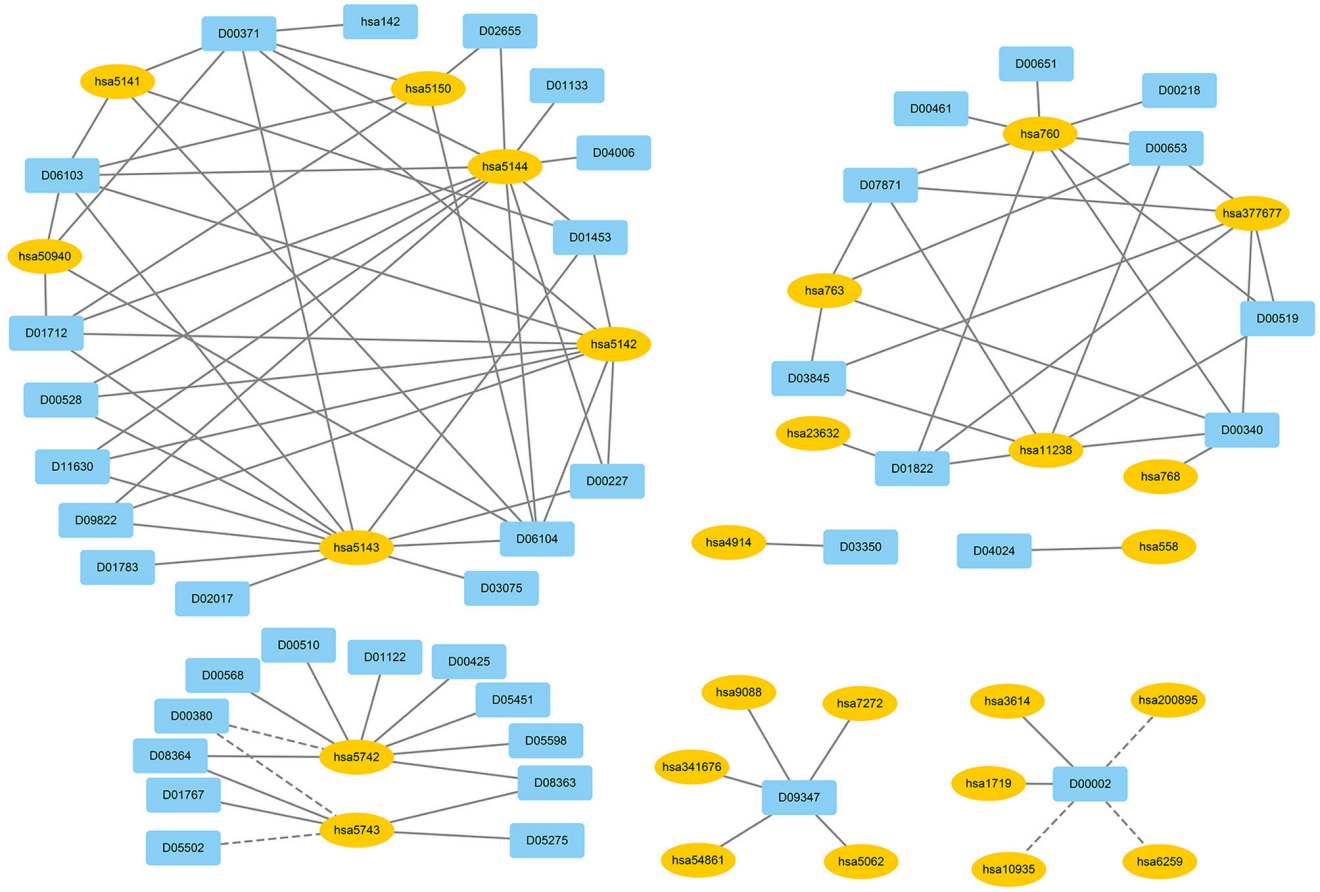
The predicted top 100 DTIs on enzymes.

We used EnGDD to find possible DTIs from unknown drug-target pairs on the four DTI datasets after confirming its performance. We first computed interaction probability for each drug-target pair based on EnGDD and ranked each drug-target pair in descending order based on the computed probabilities. Figures 5–8 list the top 100 drug-target pairs with the highest interaction probabilities on the four DTI datasets, respectively. In the figures, black solid lines and black dotted lines indicate known and unknown DTIs obtained from EnGDD, respectively. Deep sky blue diamonds and yellow ellipses denote drugs and targets.

On the nuclear receptor dataset, we predicted that D00182 and hsa2099 have a higher interaction probability among unknown drug-target pairs. D00182 (Norethisterone) is a synthetic second-generation progestin and used to protect cortical bone. Norethindrone with daily 5 or 10 mg can produce the same functions on biochemical markers of bone turnover as estrogen (DeCherney, 1993; Ferrero et al., 2010; Syed, 2022). hsa2099 (Estrogen receptor) is a nuclear hormone receptor binding to estrogen response elements with high affinity. The steroid hormones and their receptors are densely linked to cellular proliferation and differentiation in tissues (Klinge, 2001). In addition, progestins affect the bone that may be caused by stimulation of translocation relevant to the estrogen receptor and part of norethindrone is transformed to ethinyl estradiol in the rat liver (DeCherney, 1993), which further verified that D00182 and hsa2099 may interact with each other.

On the GPCR dataset, D07872 and hsa1813 were predicted to interact with a higher interaction probability. D07872 (Dosulepin) is a tricyclic antidepressant and weak inhibitor of dopamine reuptake. It interacts with many receptors (Nakagawa et al., 2000; Lepping and Menkes, 2007). It is used in patients suffering from ineffective alternative therapies because of its toxicity potential. hsa1813 is a dopamine receptor. Its activity is usually mediated by G proteins that can inhibit adenylyl cyclase (Nakagawa et al., 2000). It can be antagonized by anticancer small molecule ONC201 in clinical trials for high-grade gliomas and other cancers (Prabhu et al., 2019).

On the ion channel dataset, DB00599 and hsa2562 demonstrated a higher interaction probability. DB00599 (Thiopental) is a barbiturate. The drug can produce general anesthesia, treat convulsions, and reduce intracranial pressure (Dickinson et al., 2002). It can induce general anesthesia or complete anesthesia with short duration by intravenous administration. It is also utilized to control convulsive states for hypnosis and reduce increased intracranial pressure for neurosurgical patients (Wishart et al., 2008). hsa2562 (gamma-aminobutyric acid type A receptor subunit beta3) is a heteropentameric receptor for GABA that mainly inhibit neurotransmitter in the vertebrate brain and is also a receptor of diazepines and various anesthetics (Khair and Salvucci, 2021).

On the enzyme dataset, EnGDD predicted that D00002 may interact with hsa10935 with a higher interaction probability. D00002 (Nadide) is an important metabolic intermediate and can be used as an enzyme cofactor in redox reactions. It is involved in various enzymatic reactions as an electron carrier. It regulates various cellular functions including energy metabolism and DNA repair (Navarro et al., 2022; Pencina et al., 2023). hsa10935 (Mitochondrial peroxiredoxin 3) help to scavenge reactive oxygen species (Wang et al., 2021). It can also protect hippocampal neurons against excitotoxic injury *in vivo*. The up-regulation of peroxiredoxin-3 might be used to treat neurodegenerative diseases.

### 4.2. Target prediction for Parkinson's disease and Alzheimer's disease

In the KEGG database, D00777, D00059, D00780, D00784, D01277, D02004, D04641, D05768, D08969, D00558, D00781, D00785, D00786, and D02562 are known to be the therapeutic clues of PD (Kanehisa et al., 2017).

D00777 (Amantadine hydrochloride) is a drug only in oral formulations. It can be used to treat the PD patients. It has good absorption and little drug is present in the circulation. Associations between amantadine therapeutic effects and plasma concentrations have been confirmed by different studies (Aoki and Sitar, 1988). D00059 (Levodopa) has been validated for its “miraculous” effect in PD patients in 1961. L-dopa decarboxylase was an enzyme that can generate dopamine from levodopa. Patients with PD have a severe striatal dopamine deficit. Now, levodopa has been a “gold standard” of PD drug treatment (Hornykiewicz, 2010).

D00780 (Bromocriptine mesylate) is a dopamine receptor with an antioxidant effect (Ashhar et al., 2021). It has dopaminergic and antidyskinetic activities and is utilized to treat PD patients. Bromocriptine can selectively bind to postsynaptic dopamine D2 receptors in the central nervous system to implement the inhibition of neurotransmission and the effect of antidyskinetic (Kim et al., 2023). D00784 (Ropinirole hydrochloride) is a non-ergoline dopamine receptor agonist. It can efficiently control motor symptoms in early PD patients and has good toleration to PD (Sethi et al., 1998).

D01277 (Droxidopa) is an orally active synthetic amino acid. Neurogenic orthostatic hypotension is a fall in blood pressure on standing and notably affects PD. Droxidopa has been applied to the treatment of neurogenic orthostatic hypotension by FDA. Kaufmann et al. (2015). D02004 (Apomorphine hydrochloride) is an effective D1 and D2 dopamine agonist. It has a rapid antiparkinsonian function after subcutaneous administration and the effect is comparable with one of levodopa. Many studies suggest that Apomorphine is an effective therapeutic strategy for motor symptoms in PD (Unti et al., 2015).

D04641 (Istradefylline) is an adenosine A2A receptor antagonist. Kondo et al. (2015) detected the safety and effective of Istradefylline after administration once daily for 52 weeks in PD patients who experience wearing-off symptoms on levodopa treatment. They found that Istradefylline therapy was well-tolerated in levodopa-treated PD patients. D05768 (Rotigotine) is a non-ergolinic dopamine D3/D2/D1 receptor agonist. It administrates via a transdermal system and has been evaluated for the therapy of idiopathic PD (Reynolds et al., 2005). D08969 (Pimavanserin tartrate) is used to treat L-dopa-induced psychosis in PD. It is safe, well-tolerated and efficacious in the treatment of L-dopa-induced psychosis and doesn't worsen motor symptoms (Abbas and Roth, 2008).

Levodopa-D00558 (carbidopa) intestinal gel is used to treat advanced Levodopa-responsive PD with severe motor fluctuations and dyskinesia when other therapies fail to give satisfactory results in several countries (Wirdefeldt et al., 2016). D00781 (Entacapone) is a Chocolate-O-methyltransferase inhibitor. The addition of Entacapone in PD patients who have motor fluctuations can improve motor fluctuations (Schrag, 2005). (Mishra et al., 2019) found that D00785 (Selegiline hydrochloride) loaded nano lipid carrier administered through the nasal route has the potential for PD management therapy. D00786 (Tolcapone) may be beneficial to the PD patients who have not still developed motor fluctuations (Waters et al., 1998). D02562 (Rasagiline mesylate) is a potent and non-reversible MAO-B inhibitor. It has neuroprotective activities and a good safety and a helpful clinical effect in fluctuating PD patients who have been given an add-on to chronic levodopa therapy (Rabey et al., 2000).

The above 14 drugs are known to be therapy strategies for PD patients Table 7 gives the top 10 targets interacting with these drugs with the highest probabilities. In Table 7, each row lists a drug associated with PD and its predicted top 10 targets. The bold fonts denote that corresponding targets have no interaction with the query drug but are predicted to interact with the drug. The normal fonts denote that the query drug is known to interact with corresponding targets and the interactions are also predicted. In particular, D00059, D00780, D00784, D02004, and D05768 have been validated to interact with hsa1813 on the GPCR dataset. EnGDD predicted that D01277, D04641, and D08969 may interact with hsa1813 with the ranking of 5, 7, and 2, respectively. hsa1813 is a dopamine receptor D2. Dopamine is a neurotransmitter in the brain. Its concentration is directly associated with PD. Its low concentration in the substantia nigra can inhibit the transmission of nerve impulses and makes the brain fail to transduct signals in the proper way, which causes the brain and other body parts to lose connection (Latif et al., 2021). The above results show that D01277, D04641, and D08969 can be applied to the PD treatment by targeting hsa1813.

**Table 7:** Target prediction for Parkinson's disease and Alzheimer's disease.

**Disease**	**Dataset**	**Drug**	**Target**									
PD	IC	D00777	hsa2906	hsa2905	hsa2902	hsa2903	hsa2904	hsa116443	hsa1137	hsa1139	hsa1136	**hsa2555**
			D00780	hsa3356	hsa3357	hsa147	hsa3358	hsa1813	hsa150	hsa3350	hsa148	hsa146	hsa152
			D00784	hsa1813	hsa150	hsa148	hsa147	hsa146	hsa152	hsa151	hsa1814	hsa1815	**hsa1131**
			D01277	hsa154	hsa148	**hsa1812**	**hsa1814**	**hsa1813**	hsa153	**hsa1815**	hsa147	hsa155	hsa146
			D02004	hsa3356	hsa150	hsa1813	hsa152	hsa3358	hsa151	hsa1812	hsa3357	hsa3350	hsa1814
			D04641	**hsa3269**	**hsa1129**	**hsa3356**	**hsa148**	**hsa146**	**hsa1128**	**hsa1813**	**hsa1132**	**hsa1131**	**hsa1133**
			D05768	hsa1813	**hsa1129**	**hsa1131**	**hsa1128**	**hsa3356**	hsa1812	**hsa1132**	**hsa3269**	**hsa3358**	hsa3350
			D08969	hsa3356	**hsa1813**	**hsa3269**	**hsa147**	**hsa148**	**hsa1812**	**hsa146**	hsa3358	**hsa1815**	**hsa3350**
			D00781	hsa1312	**hsa5742**	**hsa240**	**hsa5743**	**hsa5141**	**hsa5143**	**hsa5144**	**hsa5142**	**hsa5149**	**hsa7155**
			D00785	hsa4129	hsa4128	**hsa476**	**hsa43**	**hsa1576**	**hsa5743**	**hsa5149**	**hsa5148**	**hsa5742**	**hsa590**
			D00786	**hsa5743**	**hsa5742**	**hsa240**	**hsa1728**	hsa1312	**hsa1576**	**hsa4128**	**hsa79001**	**hsa1557**	**hsa1558**
			D01277	**hsa1644**	**hsa7054**	**hsa6898**	**hsa51067**	**hsa5743**	**hsa8565**	**hsa7298**	**hsa5742**	hsa5053	**hsa7153**
			D02562	hsa4129	hsa4128	**hsa1576**	**hsa5142**	**hsa5143**	**hsa5144**	**hsa8622**	**hsa5153**	**hsa5136**	**hsa199974**
	AD	IC	D02173	hsa1137	hsa1135	hsa1139	hsa1138	hsa57053	hsa1141	hsa1143	hsa8973	hsa1136	hsa1134
					D04905	hsa2557	hsa2559	hsa2555	hsa2554	hsa2564	hsa2556	hsa2558	hsa2567	hsa55879	hsa2565
			D02173	hsa590	hsa43	**hsa5743**	**hsa1576**	**hsa7150**	**hsa5137**	**hsa5140**	**hsa5742**	**hsa5152**	**hsa3717**
			D02558	hsa43	hsa590	**hsa5742**	**hsa5743**	**hsa4842**	**hsa4919**	**hsa1559**	**hsa4843**	**hsa6476**	**hsa477**
			D03822	hsa43	hsa590	**hsa5743**	**hsa5742**	**hsa4129**	**hsa240**	**hsa112**	**hsa59272**	**hsa114**	**hsa477**

The bold value denotes the best performance in each row.

In the KEGG database, D02173, D04905, D00670, D02558, and D03822 are the therapeutic clues of AD. Table 7 gives the top 10 targets interacting with these drugs with the highest probabilities.

D02173 (Galantamine hydrobromide) is a tertiary alkaloid extracted from plants. It is now synthesized and used to treat mild to moderate AD and provides one choice of an acetylcholinesterase inhibitor for the treatment of AD (Zarotsky et al., 2003). D04905 (Memantine hydrochloride) is the first drug approved by the US FDA and used to treat moderate to severe AD (Witt et al., 2004). D00670 (Donepezil hydrochloride) is one class of AChE inhibitors that can be used for the therapy of AD. It has longer and more selective function and manageable adverse effects (Sugimoto, 2001).

Cholinesterase inhibitors are “first-line” agents used for the treatment of AD. D02558 (Rivastigmine tartrate) and donepezil (cholinesterase inhibitors) have a dose-response association. Rivastigmine tartrate is as a carbamate inhibitor of acetylcholinesterase and is used for the treatment of mild to moderate AD under the trade name of Exelon (Shamsi et al., 2020). D03822 (Rivastigmine) has been reported to improve or maintain AD patients' performance including cognitive function, global function, and behavior. Its efficacy and tolerability have been confirmed by many clinical trials (Williams et al., 2003).

The above 5 drugs are known to be the therapeutic clues of AD. In Table 7, each row lists a drug associated with AD and its predicted top 10 targets. The bold fonts denote that corresponding targets have no interaction with the query drug but are predicted to interact with the drug. The normal fonts denote that the query drug interacts with corresponding targets and the interactions are also predicted. Particularly, D02173, D02558, and D03822 are three agents used in the treatment of AD. EnGDD predicted that the three drugs may interact with hsa5743 with the ranking of 3, 4, and 3, respectively. hsa5743 is prostaglandin-endoperoxide synthase 2 (PTGS2). Xie et al. (2022) have reported that baicalein may influence the progression of AD through regulating the expression of PTGS2. Thus, D02173, D02558, and D03822 may be the clues of treatment for AD patients through targeting PTGS2.

## 5. Conclusion

In this study, we developed a computational method EnGDD for possible DTI identification. EnGDD combined feature extraction, dimensional reduction, and DTI classification with an ensemble of Grownet, DNN, and DeepForest. EnGDD obtained better performance than the other seven DTI prediction models. Parkinson's disease and Alzheimer's disease are two neurodegenerative diseases. The results from the case studies by EnGDD show that D00002 (Nadide) may be a potential drug for neurodegenerative diseases. In addition, hsa1813 and hsa5743 may be possible targets of Parkinson's disease and Alzheimer's disease, respectively.

In the future, we will design a novel deep learning model to improve DTI prediction performance and find potential drugs and targets for neurodegenerative diseases (Chen et al., 2019; Sun et al., 2022; Wang et al., 2023; Zhang et al., 2023). In addition, with the rapid development of artificial intelligence technologies, novel drug research, and development for the two diseases can be performed by molecular generation and retrosynthesis (Sridharan et al., 2022; Yu et al., 2023).

## Data availability statement

The original contributions presented in the study are included in the article/supplementary material, further inquiries can be directed to the corresponding author/s.

## Author contributions

LZ, YW, ZL, and XL: conceptualization and validation. YW and LP: methodology and data curation. LZ and YW: software. LZ and LP: investigation. YW: writing—original draft preparation and visualization. LZ, YW, and LP: writing—review and editing. LZ, ZL, and XL: supervision. LZ and XL: project administration. LZ, LP, and ZL: funding acquisition. All authors have read and agreed to the published version of the manuscript.
